# Diffuse Polyenthesitis after Intravesical Bacillus Calmette-Guerin Therapy

**DOI:** 10.31662/jmaj.2025-0019

**Published:** 2025-05-26

**Authors:** Takashi Nawata, Michihiro Toyoshige, Motoaki Sano

**Affiliations:** 1 Department of Medicine and Clinical Science, Yamaguchi University Graduate School of Medicine, Ube, Japan; 2 Department of Internal Medicine, Sanyo-Onoda Municipal Hospital, Sanyo-Onoda, Japan

**Keywords:** diffuse polyenthesitis, intravesical Bacillus Calmette-Guerin therapy, 18F-fluorodeoxyglucose positron emission tomography/computed tomography

A 72-year-old Japanese man experienced pain in the extremities and bilateral leg swelling. He had a history of bladder carcinoma and underwent intravesical Bacillus Calmette-Guerin (BCG) therapy. Two months after the last intravesical BCG therapy, the patient noticed the pain and presented at the outpatient clinic.

Laboratory examination showed elevated serum C-reactive protein levels (CRP: 1.48 mg/dL; normal range: below 0.14 mg/dL), normal serum matrix metalloproteinase-3 levels, and negative anti-citrullinated proteins antibodies and rheumatoid factor and antinuclear antibodies; 18F-fluorodeoxyglucose positron emission tomography/computed tomography showed fluorodeoxyglucose accumulation in a broad range of muscle-tendon attachment sites (Figure 1). The patient was diagnosed with diffuse polyenthesitis relevant to intravesical BCG therapy. However, 4 months after the last intravesical BCG therapy, his pain symptoms showed spontaneous remission, and serum levels of CRP decreased to 0.23 mg/dL.

**Figure 1.**
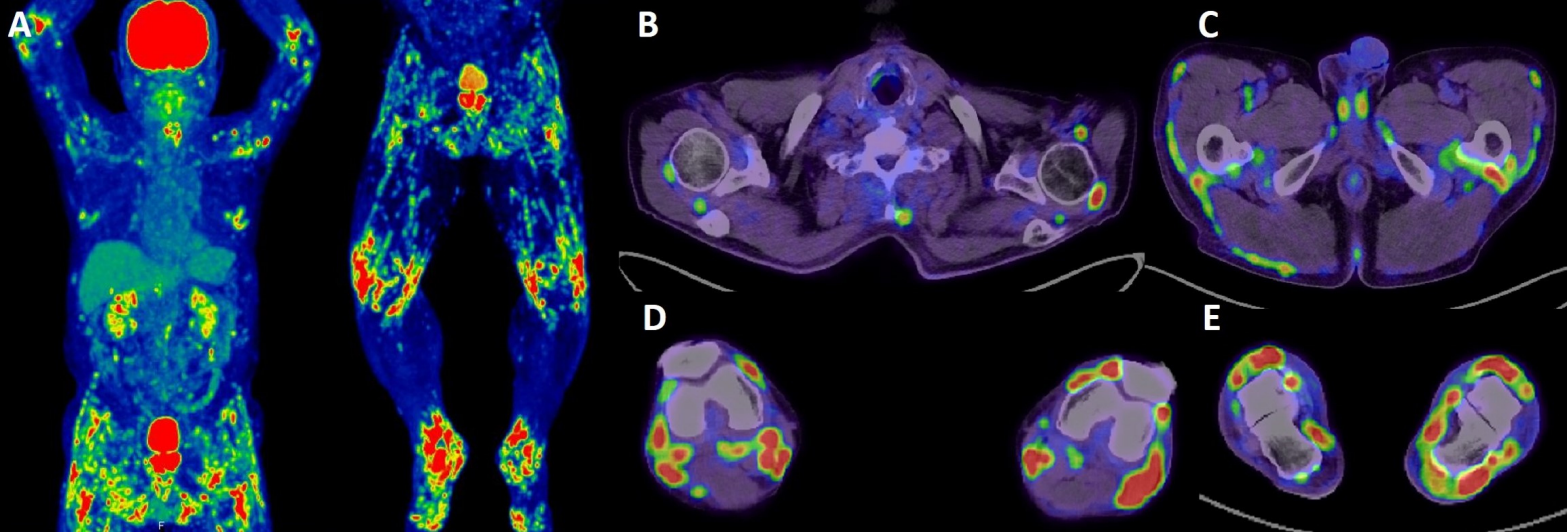
Imaging findings of the patient. This panel presents 18F-fluorodeoxyglucose positron emission tomography/computed tomography showing accumulation of fluorodeoxyglucose in numerous muscle-tendon attachment sites. (A) whole body, (B) shoulder region, (C) lumbar region, (D) knee region, and (E) foot region. SUV_mean_ ± SD of each site: attachment sites of subscapular fossa and subscapularis muscle, 3.25 ± 0.86; attachment sites of the clavicle and subclavian muscle, 2.00 ± 0.56; attachment sites of the humeral head and supraspinatus muscle, 6.85 ± 2.38; attachment sites of the humeral head and biceps brachii tendon, 4.68 ± 1.45; attachment sites of femur and gluteus minimus, 2.54 ± 0.31; attachment sites of femur and gluteus medius, 2.80 ± 0.45; attachment sites of coccyx and gluteus maximus, 5.01 ± 0.84; attachment sites of the coccyx and iliac muscle, 4.36 ± 0.71; attachment sites of the coccyx and obturatorius externus muscle, 3.46 ± 0.73; and tendon-to-bone attachment sites of knees, 12.70 ± 2.44. SD: standard deviation; SUV_mean_: mean standardized uptake value.

Our case suggests the importance of recognizing that intravesical BCG therapy can be a cause of polyenthesitis ^(1)^.

## Article Information

### Conflicts of Interest

None

### Author Contributions

Takashi Nawata was responsible for the conceptualization, methods, and writing―original draft. Michihiro Toyoshige and Motoaki Sano undertook the supervision and approved the final version to be submitted.

### Approval by Institutional Review Board (IRB)

Our institution does not require ethical approval for reporting individual cases or case series. Written informed consent was obtained from the patient for publication of this report.
